# Associated factors, diagnosis and management of *Acanthamoeba* keratitis in a referral Center in Southern China

**DOI:** 10.1186/s12886-017-0571-7

**Published:** 2017-10-02

**Authors:** Jing Zhong, Xingyi Li, Yuqing Deng, Ling Chen, Shiyou Zhou, Weilan Huang, Shiqi Lin, Jin Yuan

**Affiliations:** 1 State Key Laboratory of Ophthalmology, Zhongshan Ophthalmic Centre, Sun Yat-Sen University, Guangzhou, 510064 China; 2 Department of Ophthalmology, The Third Affiliated Hospital of Sun Yat-sen University, Guangzhou, 510630 China

**Keywords:** *Acanthamoeba* keratitis, Associated factors, Diagnosis, Keratoplasty

## Abstract

**Background:**

To analyse the associated factors, diagnosis, clinical manifestations and therapeutic effects of *Acanthamoeba* keratitis at a tertiary ophthalmic centre in Southern China.

**Methods:**

A retrospective clinical study was performed in fifteen patients who were admitted to Zhongshan Ophthalmic Centre (ZOC) from January 2004 to December 2014. The patients’ pathogenesis-associated factors were analysed, and preoperative diagnoses were determined using corneal scraping cultures and/or confocal microscopy followed. All diagnoses were confirmed by postoperative pathological examinations. At follow-up, best-corrected visual acuity (BCVA), the recurrence rate and graft transparency were evaluated to assess therapeutic effects.

**Results:**

The main pathogenic factors observed in the fifteen patients were a history of injury or a foreign body entering the eyes (12 cases). In all, *Acanthamoeba* keratitis was preoperatively diagnosed in 5 cases using corneal scraping cultures or confocal microscopy. Ocular symptoms included redness, photophobia, tearing, and blurred vision. Penetrating keratoplasty was performed in thirteen patients, and postoperative pathological examinations were performed to confirm these diagnoses. The logarithm of the minimum angle of resolution (logMAR) of visual acuity was significantly improved after keratoplasty (*p* < 0.01). No recurrence was observed, and approximately 90% of the corneal grafts were found to be transparent during the follow-up period.

**Conclusions:**

Corneal trauma may be the main pathogenic factor that causes *Acanthamoeba* keratitis in southern China. Corneal scraping combined with confocal microscopy was helpful for achieving a correct diagnosis. Early keratoplasty combined with amoebicidal therapy is an effective treatment strategy in *Acanthamoeba* keratitis.

## Background

*Acanthamoeba* keratitis (AK) is a rare infectious keratitis caused by an amoeba that is widespread in the natural environment. AK was first reported in 1973 [1], and cases are widely dispersed. AK is closely associated with contact lenses, trauma, and injury with contaminated water or soil [2, 3]. Clinically, the early stages of AK lacks characteristic clinical symptoms [1], especially in the cases involving coinfection with fungi or bacteria. These cases are often misdiagnosed and have a poor prognosis. Moreover, while some anti-amoebic drugs appear to exert greater cysticidal activity in vitro, few topical anti-amoebic agents are available because of their poor corneal penetration and topical toxicity [4]. Doctors must therefore remove the pathogens via corneal transplantation. It is difficult to achieve a diagnosis of AK, anti-amoebic drugs have poor therapeutic effects, and sources of corneal donors are limited. Hence, AK is considered a substantial challenge for ophthalmologists.

In the present study, we analysed the diagnosis and treatments of and factors associated with 15 cases of AK in southern China. These results may prompt an increase in the early diagnosis of and provide a therapeutic reference for clinical treatments for AK.

## Methods

### Patients and clinical manifestations

Fifteen patients who were diagnosed with AK at Zhongshan Ophthalmic Centre at Sun Yat-sen University between January 2004 and December 2014 were included in this study. The medical records of all included patients were reviewed to obtain the following features: age, gender, profession, predisposing associated factors, initial diagnosis and treatments applied before AK was diagnosed, duration of onset, symptoms and signs, laboratory tests, treatment, type of keratoplasty, duration of follow-up, complications and graft survival.

### Diagnostic methods

The protocol used to achieve a diagnosis of AK included confocal microscopy (HRT-RCM, Heidelberg Engineering GmBH, Dossenheim, Germany) and corneal scrapings followed by Gram-staining of cultures grown in MacConkey agar, blood agar, Sabouraud dextrose agar, and non-nutrient agar plates covered with *Escherichia coli*. Postoperatively, the corneal buttons removed during corneal transplantation were cut into several pieces and prepared for pathological biopsy and HE (haematoxylin and eosin) and periodic acid-silver metheramine PASM staining and for cultures of *Acanthamoebic*, bacteria and fungi.

### Medical and surgical treatment

Following a diagnosis of AK, the patients were administered topically neomycin (0.5%) every 2 h, propamidine (0.1%) every 2 h, fluconazole (0.2%) four times a day, clotrimazole (1%) four times a day, and fluconazole (0.5%) and clotrimazole (1%) ointment every night. The patients who responded poorly to topical therapy underwent penetrating keratoplasty. Indications for performing keratoplasty included the following: 1. an increase in the size of the corneal abscess lesion or corneal perforation; 2. persistent abscess infiltration and unbearable eye pain; and 3. no significant change in the corneal lesion accompanied by untreatable secondary glaucoma, hyphaema or other complications. Postoperatively, the patients was administered topical neomycin, propamidine, fluconazole and clotrimazole four times a day for at least 6 months [5, 6]. A solution of 0.05% Tacrolimus (FK506) was prescribed for application 4 times a day to avoid transplant rejection. The use of corticosteroids was avoided within the first month following surgery.

### Follow-up

Every patient was closely followed up. Best corrected visual acuity (BCVA), AK recurrence, graft transparency, intraocular pressure (IOP), and other complications were recorded during follow-up. The final BCVA was defined as the best vision obtained preoperatively, 2 weeks postoperatively, and at the last visit after surgery. Snellen visual acuity was recorded, and approximations for visual acuity worse than 20/400 were determined as follows: counting fingers = 20/2000, hand motions = 20/4000, light perception = 20/8000 and no light perception = 20/16000. Snellen vision was converted to logMAR for statistical analysis [7]. The following criteria were used to define recurrence of AK were: a purulent corneal ulcer that re-appeared in the graft and positive confocal microscopy findings or positive corneal biopsy or histopathological confirmation if a repeat keratoplasty was performed. Graft failure was defined as the irreversible loss of central graft clarity and the loss of visual acuity. For eyes in which the graft did not remain clear, the follow-up interval included the period from surgery to graft failure [8, 9].

## Results

### Patients and clinical manifestations

We examined 15 eyes in fifteen patients with AK. Of these, 10 (10/15, 66.67%) were males, and the mean age was 42.27 ± 13.35 (19–63) years old. Most of the patients were poor (9/15, 60%). Five patients (5/15, 33.33%) had a history of injury. Of these, Patient (P) 5 (P5) was injured by an iron wire and had shallow central corneal stromal wounds; P12, P13 and P14 were hit by a stick and had some corneal epithelial damage; and P15 had been stabbed by the tip of pencil and had small but deep corneal stromal wounds. Seven (7/15, 46.67%) of the patients had been recently exposed to contaminated water/insects/foreign bodies. Only one case (1/15, 6.67%) had a history of wearing contact lenses. The time from the onset of infection to the initial consultation at our hospital was 52 ± 25.29 (range 20–120) days (Table 1).

**Table 1:** General information and clinical diagnoses in the included patients

Case no.	Sex	Age (y)	Profession	Eye	Risk Factor	Disease course	Initial diagnosis
P1	Female	55	Actress	Left	Eye exposure to cosmetics	4 m	AK
P2	Male	44	Fisherman	Left	Eye exposure to fish pond water	20d	AK
P3	Male	60	Farmer	Right	Mosquito entering into the eye	2 m	FK
P4	Female	23	Clerk	Right	Wearing contact lenses	1 m	HSK
P5	Male	52	Farmer	Left	Hit with an iron wire	2 m	FK
P6	Male	49	Farmer	Left	Eye exposed to paddy field water	45d	AK
P7	Female	59	Farmer	Right	Winged insect in the eye	1 m	AK
P8	Male	39	Upfitter	Right	Eye exposed to cement	2 m	FK
P9	Male	28	Fisherman	Left	Unclear	40d	HSK
P10	Male	37	Farmer	Right	Unclear	1 m	BK
P11	Male	19	Worker	Right	Eye exposed to foul water	1 m	AK
P12	Male	63	Farmer	Left	Injured by a stick	2 m	FK
P13	Male	37	Farmer	Right	Injured by a stick	1.5 m	FK
P14	Female	39	Farmer	Left	Injured by a stick	3 m	FK
P15	Female	30	Worker	Right	Injured by a pencil	2 m	FK

P1 = Patient 1, P2 = Patient 2…P15 = Patient 15; *AK Acanthamoeba* keratitis, *FK* fungal keratitis, *BK* bacterial keratitis, *HSK* herpes simplex keratitis

**Fig. 1:**
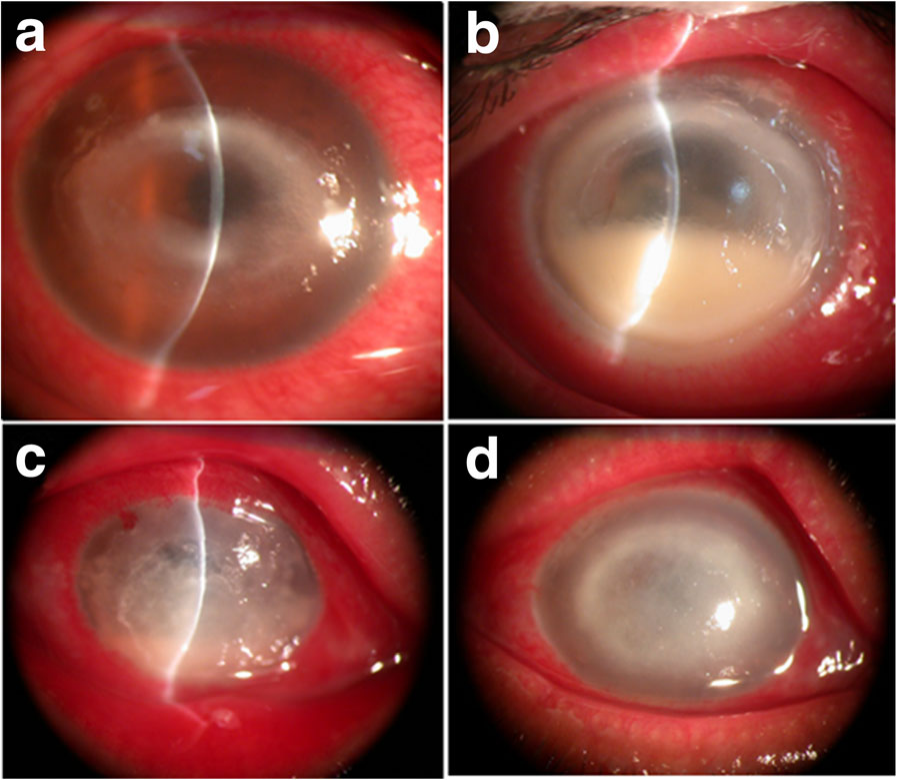
*Acanthamoeba* keratitis is characterized by ring-like stromal infiltrates and corneal lesions (**a**). The cornea is relatively translucent in the centre of the ring infiltrates (**b**). Coinfection with fungi or bacteria contributes to variability and atypical symptoms. A cornea was infected with *Acanthamoeba* and *Aspergillus* (**c**). A cornea infected with *Acanthamoeba*, *Mucor* and *E. coli* (**d**)

All patients had unilateral involvement. The main complaints were redness, photophobia, tearing, and blurred vision. Five patients presented with ring infiltration, 8 with multifocal stromal infiltration, and 2 with corneal perforation. No radial keratoneuritis or posterior segment involvement was observed (Fig. 1).

### Microbiology, confocal microscopy, and histopathology

Among the 15 included patients, confocal microscopy and corneal scraping cultures revealed *Acanthamoeba* cysts in 5 patients (5/15, 33.33%). Among these 5 patients, corneal scraping cultures confirmed that 2 were con-infected with bacteria (1 with *Mucor* species and *Escherichia coli* and the other with *Aspergillus fumigatus*) (Table 2). Ten patients (10/15, 66.67%) had been misdiagnosed with fungal infections (7 cases), herpes simplex keratitis (2 cases), or bacterial keratitis (1 case). These patients had been treated with topical and/or systemic antifungal, antiviral, or antibiotic drugs, respectively.

**Table 2:** Laboratory tests performed in the patients

Case no.	Confocal Scan (pre-surgery)	Microbiologic smear and culture (pre-surgery)	Histopathology (post-surgery)
P1	Amoebic cysts	*Acanthamoeba* and *Staphylococcus epidermidis*	(−)
P2	Amoebic cysts	*Acanthamoeba*, *Mucor* and *Escherichia coli*	*Acanthamoeba*, *Mucor* and *Escherichia coli*
P3	(−)	(−)	*Acanthamoeba*
P4	(−)	(−)	*Acanthamoeba*
P5	(−)	(−)	*Acanthamoeba*
P6	Amoebic trophozoites	*Acanthamoeba* and *Aspergillus fumigatus*	*Acanthamoeba* and *Aspergillus fumigatus*
P7	Amoebic trophozoites	*Acanthamoeba* and *Staphylococcus* epidermidis	*Acanthamoeba* and *Staphylococcus* epidermidis
P8	(−)	(−)	*Acanthamoeba*
P9	(−)	(−)	*Acanthamoeba*
P10	(−)	(−)	*Acanthamoeba*
P11	Amoebic trophozoites	(−)	(−)
P12	(−)	(−)	*Acanthamoeba*
P13	(−)	(−)	*Acanthamoeba*
P14	(−)	(−)	*Acanthamoeba*
P15	(−)	(−)	*Acanthamoeba*

**Fig. 2:**
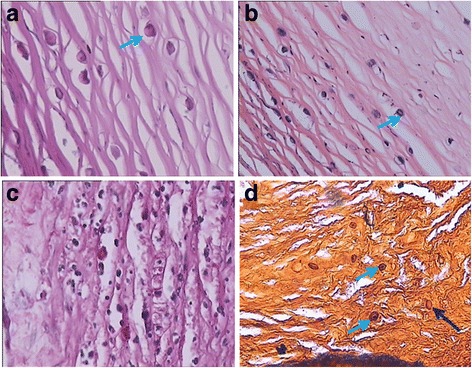
Pathological sections stained with HE revealed corneal oedema and amoebic cysts among the corneal collagenous fibres (blue arrow) (**a**-**b**). Numerous polymorphonuclear and mononucleated cells and nuclear debris had infiltrated the corneal stroma (**c**). Amoebic cysts and trophozoites were detected in the cornea (blue arrow) using PASM staining. *Aspergillus* hyphae (deep blue arrow) had fragmented in a coinfected case (**d**) (20 × 20)

The 13 patients who underwent penetrating keratoplasty were confirmed by HE and PASM staining. HE staining (Fig. 2a and b) showed that the *Acanthamoeba*-infected corneas exhibited serious corneal stromal oedema and amoebic cysts among corneal collagenous fibres. Moreover, numerous polymorphonuclear and mononucleated cells and nuclear debris had infiltrated into the corneal stroma (Fig. 2c). PASM staining showed indicated the presence of amoebic cysts and trophozoites in the cornea, and *Aspergillus* hyphae were found to have fragmented in the case coinfected with *Acanthamoeba* and *Aspergillus fumigatus* (Fig. 2d).

### Postmedical treatment results

Topical antiamoebic treatments, including neomycin, propamidine, fluconazole and clotrimazole, were used in all patients after diagnosis. The patient who was coinfected with *Acanthamoeba* and a fungus was additionally treated with topical natamycin (5%) every 2 h. The patients with concomitant bacterial infection were topically treated with levofloxacin (0.5%) every 2 h. Two cases (P1 and P11) received only medical therapy without surgery. After 6 months, in one of these patients (P1), the corneal epithelium recovered and ulceration resolved with scarring, while in the other patient (P11), the corneal infiltrates and ulcerations neither improved nor worsened.

### Postoperative treatment results

**Fig. 3:**
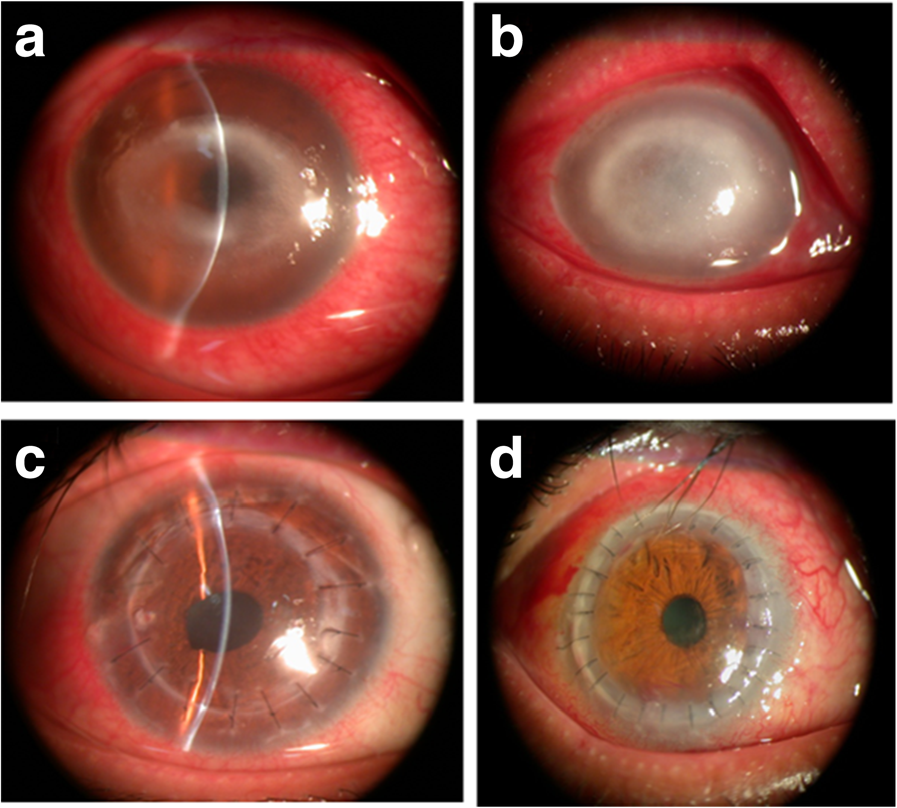
A moderately infected case (**a**) that was treated with conventional penetrating keratoplasty. The graft was transparent, and the depth of the anterior chamber was normal after six months (**b**). A seriously infected case (**c**) that was treated with large corneo-scleral penetrating keratoplasty. The donor cornea was clear after one month (**d**)

The thirteen patients (P2 to P10 and P12 to P15) who underwent penetrating keratoplasty were followed up for 6 to 36 months (mean, 30 months). None of these patients experienced a recurrence of AK during the follow-up period. Early postsurgical hyphema occurred in four cases, only one of which required anterior chamber irrigation. Intraocular pressure increased in three patients during the follow-up period and was successfully controlled medically. Graft failure occurred after 1 year in three patients (3/13, 23.08%). One of these patients underwent repeated keratoplasty, and the graft remained clear through the last follow-up. The grafts implanted in the other ten patients remained clear during the follow-up period (Fig. 3).

**Fig. 4:**
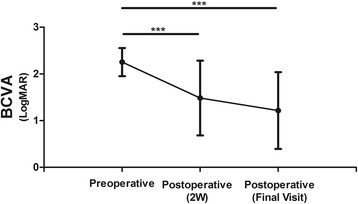
Pre- and post-operative logMAR best corrected visual acuity of the patients. The data are shown as the mean ± SEM. *, *p* < 0.05; **, p < 0.01; and ***, p < 0.001

The LogMAR BCVA was 2.255 ± 0.089 and improved to 1.483 ± 0.240 at 2 weeks postoperatively (*p* < 0.01). The final LogMAR visual outcome after keratoplasty was 1.040 ± 0.262 (*p* < 0.001). These results show that BCVA was significantly improved in these patients (Table 3 and Fig. 4).

**Table 3:** Preoperative condition and postoperative follow-up data

Case no.	Preoperative vision lo	Size of lesion (mm)	Surgery	Postoperative BCVA (2w)	Postoperative BCVA (final visit)	Complications
P2	2.301 (20/4000)	8	PKP	1.602 (5/200)	1.301(10/200)	Ocular hypertension; Hyphaema
P3	2.301 (20/4000)	7	PKP	0.477 (20/60)	2.301 (20/4000)	Ocular hypertension; Hyphaema; Corneal graft
P4	2.301 (20/4000)	5	PKP	0.400 (20/50)	0.400 (20/50)	–
P5	2.301 (20/4000)	8	PKP	2.301 (20/4000)	1.602 (5/200)	Hyphaema
P6	1.380 (8/200)	3	PKP	1.000 (20/200)	0.400 (20/50)	–
P7	2.602 (20/8000)	8	PKP	2.602 (20/8000)	2.602(20/8000)	Corneal graft rejection
P8	2.602 (20/8000)	Perforation	PKP	2.602 (20/8000)	2.301 (20/4000)	Ocular hypertension; Hyphaema
P9	2.301 (20/4000)	9	PKP	0.700 (20/100)	0.700 (20/100)	Corneal graft rejection; Second set transplantation
P10	2.602 (20/8000)	Perforation	PKP	2.602 (20/8000)	2.301 (20/4000)	–
P12	2.000 (20/2000)	7	PKP	0.700 (20/100)	0.400 (20/50)	–
P13	2.000 (20/2000)	8	PKP	1.602 (5/200)	0.700 (20/100)	–
P14	2.301 (20/4000)	8	PKP	2.000 (20/2000)	0.400 (20/50)	–
P15	2.301 (20/4000)	6	PKP	0.700 (20/100)	0.400 (20/50)	Transient ocular hypertension

## Discussion

*Acanthamoeba* keratitis was first reported in 1973. Because amoebae have a widespread distribution throughout the natural environment and contact lens use is increasing, the incidence of AK has gradually increased over the last 20 years [1]. In developed countries, most AK patients are contact lens wearers. The estimated national incidence of contact lens use varies widely from 1.65–2.01/million people in the United States to 17.53–19.50/million people in the United Kingdom [10]. A small number of AK cases have been reported to be caused by trauma, exposure to contaminated water or soil, or other reasons [11, 12]. In China, the factors associated with AK include trauma, soil-contaminated matter and contact lens use, especially orthokeratology, as confirmed by a study performed by Erdem and Chan [13, 14]. However, more than half of cases are caused by trauma or exposure to contaminated water when the rate of contact lens users is approximately 0–30%, a result that is quite different from that observed in developed countries. In our study, seven (7/15, 47%) patients were exposed to contaminated water/insects/a foreign body, five (5/15, 33%) had a history of minor trauma, and one (1/15, 7%) was a contact lens wearer. No definite risk factor was identified in two (2/15, 13%) cases. Of the 15 included patients, 9 were peasants, 4 were labourers, 1 was a secretary, and 1 was an actress. Most lived in poor sanitary conditions, which increases the risk of exposure to pollutants and eye trauma.

In its early stage, AK can resemble pseudodendriform keratitis and can be easily misdiagnosed as herpes simplex virus keratitis. The perineural infiltration of amoeba trophozoites can lead to radial neuritis at any stage of the disease and is observed in 29% to 63% of affected patients [15]. The most typical symptom during the later period is a ring infiltration lesion in the corneal stroma. This is, according to a report by Bacon et al., more commonly associated with the late than the early stage of the disease [16]. In our study, all of the patients complained of ocular symptoms, including redness, photophobia, tearing, and blurred vision, while clinical signs were more variable. These patients did not display ring infiltration, radial keratoneuritis or other typical signs. Moreover, four of the patients who were co-infected with bacterial and fungi presented with more atypical symptoms, which increased the difficulty of achieving an early clinical diagnosis and resulted in a rate of misdiagnosis as high as 66.7% (10/15). It was therefore difficult to achieve a diagnosis based on clinical signs alone, and confocal microscopy, corneal scraping cultures, histology, and PCR are regarded as helpful for accurately diagnosing AK [17–19].

Confocal microscopy is considered the best non-invasive diagnostic technique for treating amoebic keratitis (its sensitivity and specificity exceed 90% for individual unmasked observers) [20–22]. Auran et al. first reported using confocal microscopy as a diagnostic technique for amoebic keratitis in 1994 [18]. Parmar reported a group of cases of AK in 2005 and found that positive rate was 76% when confocal microscopy was used [23]. In the current study, all of the patients were analysed using confocal microscopy, resulting in 5 positive cases (5/15, 33.33%). We speculate that this low sensitivity was mainly because most of our cases had a longer course and exhibited severe suppuration, multifocal stromal infiltration, and poor transparency on the confocal microscopy scans. Moreover, a lack of familiarity with confocal scanning may also have contributed to the low rate of positivity for amoebic cysts [24–26]. Hence, we suggest that visiting a clinician early in the disease course is helpful for achieving an early diagnosis and that confocal microscopy should be performed by a skilled operator who can produce repeatable results.

Interestingly, four of the cases (4/15, 26.67%) in this study were diagnosed with a coinfection of *Acanthamoeba* and fungi or bacteria. Parmar reported that 1 of 63 AK patients was coinfected with *Acanthamoeba* and fungus [23], Sharma published a case study in which a patient with contact lens-associated keratitis was coinfected with *Acanthamoeba* and *Pseudomonas* [27]. We suspect that trauma or the entry of a foreign body into the eyes may break the corneal immunological barrier that normally prevents humans from being infected with *Acanthamoeba* and other microbial agents. The bacterial or fungal infection destroys the microenvironment of the ocular surface, which allows amoeba to more easily penetrate the tissues [28].

Research has shown that biguanides and diamidines effectively kill cysts. However, poor therapeutic effects have been observed in severe cases, and their ocular surface toxicity limits their usefulness as anti-amoebic drugs in a clinical setting [29–31]. In our series, 13 patients underwent penetrating keratoplasty. In keratoplasty, the corneal button should be 1 mm larger than the lesions, and fluconazole solution (0.2%) is used to irrigate the anterior chamber [12, 32]. Large corneo-scleral transplantations were performed in patients in whom the lesion was too large to be treated using conventional keratoplasty. *Acanthamoeba* can only barely penetrate Descemet’s membrane to reach the anterior chamber, and a robust neutrophil response in the anterior chamber is associated with the disappearance of intraocular trophozoites. This prevents AK from progressing to endophthalmitis [33]. Therefore, penetrating keratoplasty must completely remove the *Acanthamoeba* to fully cure the eye and avoid recurrence.

None of the patients in this study suffered a recurrence during the follow-up period, whereas there were 2 recurrences in 21 cases in Dart J’s studies. We suggest that the lack of recurrence in this study might have been because PKP requires a larger corneal graft, and timely anti-amoebic therapy was performed to prevent a poor prognosis [34]. Moreover, the sample size in our study was smaller than that in Dart J’s study. Three of our cases (3/13, 23.08%) suffered graft failure, whereas in Renata’s study, 56.2% of the patients with AK suffered graft failure after therapeutic penetrating keratoplasty, and in Kitzmann’s study, 59% suffered graft failure [35]. The lower rate of graft failure observed in our study was because FK506 was prescribed postoperatively whereas corticosteroids were avoided. The role of topical corticosteroids in postoperative recovery from AK remains controversial because on the one hand they are useful for treating severe corneal inflammation, neovascularization and scleritis, while on the other hand, they also potentiate infection and are therefore associated with a poor prognosis [36]. Hence, FK506 is considered a better choice because it suppresses the expression of T-cell-mediated lymphokines and the interleukin-2 receptor and the generation of cytotoxic T cells, which effectively prevents graft rejection and maintain the transparency of the graft [37]. However, this group of patients did not achieve ideal visual outcomes, potentially because of the severe condition of the disease and the large lesions these patients presented with at our hospital. Additionally, postoperative complications of cataract surgery can result in poor visual acuity. We therefore suggest that if a patient’s response to anti-amoebic drugs is poor, therapeutic keratoplasty should be performed as soon as a clear diagnosis is achieved.

## Conclusion

Our results demonstrate that the entry of contaminated water or soil or insects into the eye and trauma are the major risk factors for *Acanthamoeba* keratitis. Confocal microscopy should play an important role in the diagnosis of these patients, an early and prompt diagnosis is associated with a good prognosis, and therapeutic keratoplasty is an effective therapy for treating AK. Our data reflect our meaningful clinical experience and represent a reference for *Acanthamoeba* keratitis.

## Abbreviations

AK: *Acanthamoeba* keratitis
BCVA: best-corrected visual acuity
FK506: Tacrolimus
HE: haematoxylin and eosin
LogMAR: Logarithm of the minimum angle of resolution
PASM: periodic Acid-Silver Metheramine
ZOC: Zhongshan Ophthalmic Centre
